# The evolution of robotics: research and application progress of dental implant robotic systems

**DOI:** 10.1038/s41368-024-00296-x

**Published:** 2024-04-08

**Authors:** Chen Liu, Yuchen Liu, Rui Xie, Zhiwen Li, Shizhu Bai, Yimin Zhao

**Affiliations:** 1 State Key Laboratory of Oral & Maxillofacial Reconstruction and Regeneration, Xi’an, China; 2 National Clinical Research Center for Oral Diseases, Xi’an, China; 3 Shaanxi Key Laboratory of Stomatology, Xi’an, China; 4 Digital Center, School of Stomatology, The Fourth Military Medical University, Xi’an, China

**Keywords:** Medical research, Preclinical research, Oral diseases

## Abstract

The use of robots to augment human capabilities and assist in work has long been an aspiration. Robotics has been developing since the 1960s when the first industrial robot was introduced. As technology has advanced, robotic-assisted surgery has shown numerous advantages, including more precision, efficiency, minimal invasiveness, and safety than is possible with conventional techniques, which are research hotspots and cutting-edge trends. This article reviewed the history of medical robot development and seminal research papers about current research progress. Taking the autonomous dental implant robotic system as an example, the advantages and prospects of medical robotic systems would be discussed which would provide a reference for future research.

The development of medical robots has been a long journey of exploration. After being practically validated in industrial robots, this technology has become widespread globally and is now an essential part of modern production and lifestyles. Medical robots are increasingly in the vanguard of the field in diagnosis, treatment, visualization, and other areas of clinical practice. We are currently witnessing a transformative shift from cutting-edge research to the widespread application of medical robots. This review focused on the historical trajectory of medical robots, with a particular emphasis on the development history, current research status, and prospects of dental implant robotic systems.

## Definition and history of robots

### Definition and architectures of robots

According to the International Organization for Standardization (ISO), a robot is an automatic, position-controlled, programmable multi-functional manipulator with several axes. It can process various materials, parts, tools, and special devices through programmable automation to perform intended tasks.^1^ A robot’s structure typically consists of four parts: the actuation system, the drive-transmission system, the control system, and the intelligent system. The actuation system is the part of the robot that directly performs work, similar to a human hand. The drive-transmission system transmits force and motion to the actuator through a power source. The control system comprises a control computer, control software, and servo controllers, similar to a human brain. The intelligent system typically includes a perception system and an analytical decision-making intelligent system.

### Evolution of robots

The history of robots can be traced back over 3 000 years.^2^ Throughout history, scientists and craftsmen have designed and manufactured robot prototypes that simulate animal or human characteristics.^1^ However, these inventions can only be classified as mechanical devices that primarily achieved automated functions through mechanical and physical principles with the lack of intelligence and autonomy of modern robots. These inventions demonstrate the level of engineering technology and mechanical manufacturing in ancient times, laying the foundation for later research on robots. Joseph Engelberger, recognized as the Father of Robotics, founded Unimation Corporation in 1958, the world’s first robot-manufacturing factory, which marked the official start of the industrialization of robots. In 1978, Unimation developed a Programmable Universal Machine for Assembly (PUMA) which represents a significant milestone in the development of international industrial robotics. In recent years, robotics has expanded significantly due to the continued development of sensor types, intelligent algorithms, and multidisciplinary integration. The technology has advanced from the initial industrial robotic arms to bionic robots, soft robots, nanorobots, and other forms.

### Classification of robotics

**Fig. 1:**
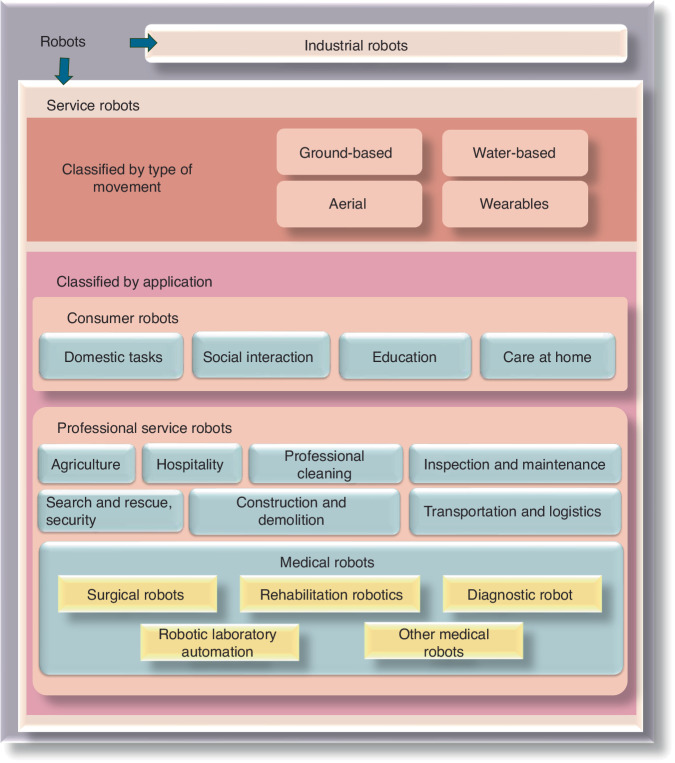
Categories of robots according to the International Federation of Robotics

The International Federation of Robotics (IFR) classifies robotics into two distinct categories: industrial robotics and service robotics, in accordance with the international standard ISO 8373:2012.^3^ Industrial robotics are multipurpose manipulators with automatic control and programmability, which can operate with fixed or autonomous mobility and are primarily used in industrial production.^3^ Service robotics are driving mechanisms that can perform useful tasks but do not include industrial automation applications. The IFR has classified service robotics into different segments to meet the diverse requirements of various industries (Fig. 1).

## Medical robotics

In 1985, the Puma 200 robot (Westinghouse Electric, Pittsburgh, PA) was used for needle placement in computed tomography (CT)-guided brain biopsy at the Los Angeles Hospital in the United States, marking the beginning of the era of medical robot applications.^4,5^ After nearly 40 years of continuous development and progress, medical robotics have been widely used in multiple fields, including surgery, nursing, and rehabilitation, demonstrating numerous remarkable advantages and potential.

Yang^6–8^ has divided the level of autonomy of medical robotics into six levels, as follows: (0) no autonomy, (1) robot assistance, (2) task autonomy, (3) conditional autonomy, (4) high autonomy, and (5) full autonomy. At level 0, the robot requires operators to perform all tasks, including monitoring, generating performance options, selecting the option to perform (decision making), and executing the decision made, such as the da Vinci robotic system (Intuitive Inc., California, USA). At level 1, operators are required to continuously control the robot while the robot provides guidance with positional constraints. The Mako Smart Robotics used in orthopedic surgery is an example. At level 2, operators are required to discretely rather than continuously control the robot, and the robot can independently complete specific tasks based on operator instructions and pre-programmed procedures. An example of this level is the ROBODOC, which performs total hip and total knee replacement surgeries. At level 3, robots have the ability to perform surgeries based on pre-programmed procedures and can also modify the pre-planned schedule in real time to accommodate changes in the intraoperative position of the target object. An example of such robotics is the CyberKnife radiation therapy robotics, which has respiratory tracking functionality. At the higher levels of autonomy (specifically level 5 and possibly level 4), the robot is not only a medical device but also capable of practicing medicine, which currently does not exist due to some regulatory, ethical, and legal considerations.^6–8^

Medical robotics are classified by IFR as special robotics with a combination of medical diagnosis methods with new technologies, such as artificial intelligence (AI) and big data, to provide services such as surgery, rehabilitation, nursing, medical transportation, and consultation for patients.^9^ Medical robotics are categorized into the following five types based on their functions: surgical robotics, rehabilitation robotics, diagnostic robotics, laboratory analysis automation, and other robotics (robotics used for medical transportation are not included in this category).

### Surgical robotics

**Fig. 2:**
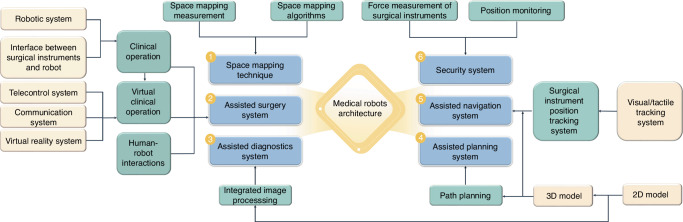
The surgical robotic architecture

Minimally invasive surgery and accurate intervention require surgeons to exercise more discernment, expand their range of vision, and increase their flexibility which brings the surgical robotics development (the surgical robot architecture^10^ was shown in Fig. 2). Not only can it be equipped with an advanced three-dimensional (3D) imaging system and augmented reality technology to provide high-definition images of the surgical scene, but it is also capable of displaying important anatomical structures such as blood vessel and nerve locations in real-time. This allows surgeons to perform precise operations with the assistance of robots. For higher-level automatic medical robots, precise surgical operations are performed through image guidance and navigation systems based on preoperative planning. Moreover, the robotic arm has a high level of precision and stability that surpasses the capabilities of a free hand. This allows it to perform small and delicate operations with reduced errors caused by physician experience, fatigue, and hand tremors. In addition, the surgical robot also integrates artificial intelligence technology, which can perform automatic diagnostic analysis, adjust surgical strategies, and provide personalized surgical plans through deep learning.^11^ Therefore, surgical robots could utilize vision, speech recognition, telecommunication, 3D imaging, and artificial intelligence technologies to enhance surgical skills through sensing and image guidance systems. This overcomes the limitations of manual operations and improves surgical accuracy and reliability. In comparison to conventional surgery, robotic-assisted surgery could reduce trauma, shorten recovery periods, and relieve pain.^12,13^ Additionally, it can be used for remote surgery, operates continuously without fatigue, reduces the workload of medical staff, and minimizes occupational exposure for surgeons. Medical robotics have gradually entered the commercialization stage and have been utilized in clinical settings (Table 1). Currently, the most well-known surgical robot is the da Vinci system, which enables surgeons to accurately and minimally perform invasive surgery for multiple complicated diseases with good hand-eye coordination and magnification.

**Table 1:** Representative commercial surgical robotics

Name of robotics	Company	Application field
NeuroMate^101^	Renishaw	Neurosurgery
ROBODOC^102^	Curexo Technology.	Orthopedics
Aesop^103^	Computer Motion Inc.	Laparoscopy
Zeus^104,105^	Computer Motion Inc.	Laparoscopy
da Vinci^106^	Intuitive Surgical Inc.	Laparoscopy
Acrobot^107^	Stryker	Orthopedics
CyberKnife^108^	Accuray Inc	Radiation therapy
SpineAssist^109^	Mazor Robotics	Spine surgery
Sensei^110^	Hansen Medical	Vascular surgery
Mako^111^	Stryker	Orthopedics
Viky^112^	Endocontrol Medical	Laparoscopy
Yomi^113^	Neocis Inc.	Dental implantation
Magellan^114^	Hansen Medical	Vascular surgery
BlueBelt Navio^115^	Smith & Nephew	Orthopedics
Yakebot^116^	Yekebot Technology Co.	Dental implantation
Flex^117^	Medrobotics	Endoluminal surgery

**Fig. 3:**
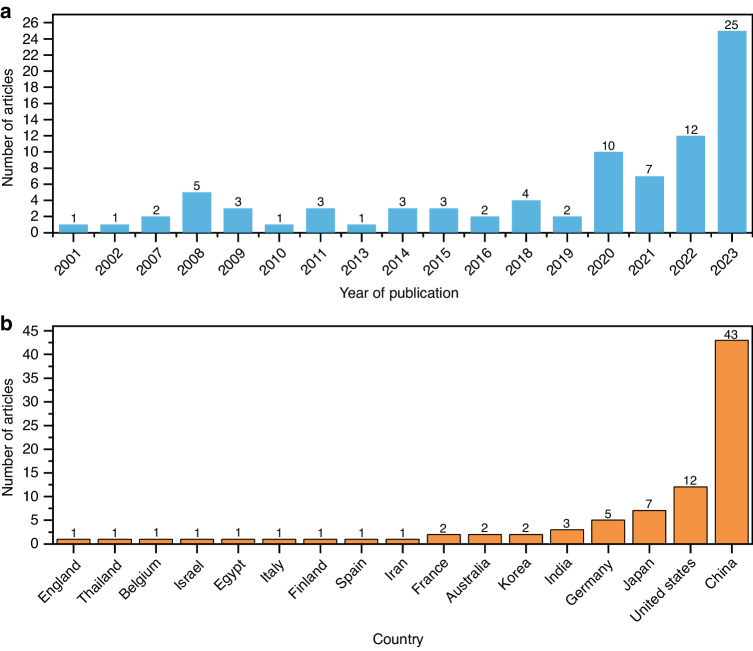
Related research on robotic-assisted dental implant placement. **a** The number of published papers on dental implant robotics in different years and **b** in different countries (as of December 2023)

Dental treatment involves the special anatomical structure of the mouth and is characterized by limited visibility, narrow operation space, and the disturbance of saliva and tongue. As a result, the dental operation is intricate and mainly reliant on the surgeon’s experience and expertize, which takes inexperienced surgeons a long time to acquire. With the successful use of the da Vinci robotic system in laparoscopic surgery, surgeons are beginning to consider its potential application in maxillofacial surgery. Da Vinci robot has been used for cleft palate repair,^14,15^ treating patients with obstructive sleep apnea-hypopnea syndrome (OSAHS),^16^ as well as oral and oropharyngeal tumor resection.^17,18^ However, due to the complexity of the oropharyngeal anatomy, the multiple robotic arms of the da Vinci system limit the surgeon’s vision, which is not conducive to surgical performance. In order to overcome these shortcomings, flexible robots (such as The Flex) approved by the Food and Drug Administration have made it possible to be used for oropharyngeal surgery. Additionally, oral and cranio-maxillofacial bone surgery, such as orthognathic surgery and dental implant surgery, requires accurate ostomies, which cannot be achieved by the da Vinci system. Robotic-assisted dental implant surgery research originated in 2001, and related studies have shown a gradual increase in recent years. In addition to conventional implant surgery, dental implant robotics can also perform zygomatic implant placement.^19,20^ Among these studies, the largest number of articles were published in China, followed by the United States (Fig. 3). In Part 3 of this article, the relevant studies on dental implant robotics will be elaborated in detail.

### Rehabilitation robotics

Rehabilitation robotics are significant area and research hotspot in medical robotics, second only to surgical robotics. Rehabilitation robotics are classified into two categories, as follows: therapeutic and assistive robotics. Therapeutic robotics provide psychological or physical treatment to improve specific functions of patients and are widely used in physical training and functional recovery of patients with paralysis and in improving the interactive ability of children with autism through behavioral induction.^21^ Assistive robotics aim to improve the quality of life for individuals with musculoskeletal or neuromuscular impairments by compensating for or replacing their mobility or functionality.^22–24^ For instance, Mike Topping’s Handy1 assists the most severely disabled with several everyday functions.^25^ Similarly, Israel’s ReWalk provides powered hip and knee motion to enable individuals with spinal cord injury to stand upright, walk, turn, climb, and descend stairs.^26^ Moreover, Japan’s wearable powered prosthesis, HAL, can enable patients to control joint movements independently by detecting bioelectrical signals on the skin surface during movement, in combination with foot pressure sensors.^27^

### Diagnostic robotics

Diagnostic robotics aid doctors in conducting examinations and making diagnoses, with the aim of improving accuracy, convenience, non-invasiveness, and safety of diagnosis. For instance, wireless capsule endoscopy introduced by Given Imaging (now Medtronic) allows minimally invasive inspection of the gastrointestinal tract. Patients can swallow a pillcam that captures images deep within the intestines, which has revolutionized gastrointestinal endoscopy and is now a clinically viable alternative to standard interventional endoscopy. Furthermore, wearable robotics are increasingly being utilized to non-invasively detect various health indicators and assist in disease diagnosis.

### Laboratory robotics

Laboratory robotics handle and analyze samples in medical laboratories. Innovations in robotics and information technologies have created new opportunities for laboratory automation. These robots tirelessly and accurately perform tasks, improving the precision and reliability of experiments while reducing costs. At the University of Virginia Medical Center, robots operate instruments and analyze blood gases and electrolytes in the hospital laboratory. In addition, the robotic system works continuously, not only improving laboratory efficiency but also reducing the burden on laboratory techniques.^28^ Nicole Rupp, based in Germany, has utilized the Dobot Magician robot to develop an economical automated laboratory system that coordinates various instruments for experiments. The results obtained from this system were not statistically different from those obtained from manual experiments.^29^

### Other medical robotics

The medical field has witnessed a significant increase in the use of robotics, leading to the development of new types of robots and functions to cater to the requirements of doctors and patients. Other medical robotics include providing non-medical operational services, such as assisting nurses with guidance, transportation, cleaning, inspection, monitoring, and disinfection. Moreover, robotics could be available for daily home care, providing assistance, monitoring behavior and health, as well as offering companionship for older individuals.^30^ Furthermore, there are robots specifically designed to train emergency personnel. These robots can simulate complex trauma scenarios with multiple injuries in a highly accurate manner.^31^ Robotic surgery simulation practice can be combined with virtual reality (VR), 3D-printed organ tissue models, or anesthetized live animals to rapidly improve the robotic surgical skills required by novice surgeons. In addition, to pandemics such as Ebola and COVID-19, the use of sampling robotics can effectively reduce the risk of infection. There are robots also designed for emergency rescue, medical education, and training.^32,33^ Soft robotics, bionic robotics, nanorobots, and other robotics suitable for various functional needs are also hot topics in current medical robotic research, and they exhibit the typical characteristics of specialization, personalization, remoteness, intelligence, and immersion.

## Dental implant robotic system

Implantology is widely considered the preferred treatment for patients with partial or complete edentulous arches.^34,35^ The success of the surgery in achieving good esthetic and functional outcomes is directly related to correct and prosthetically-driven implant placement.^36^ Accurate implant placement is crucial to avoid potential complications such as excessive lateral forces, prosthetic misalignment, food impaction, secondary bone resorption, and peri-implantitis.^37^ Any deviation during the implant placement can result in damage to the surrounding blood vessels, nerves, and adjacent tooth roots and even cause sinus perforation.^38^ Therefore, preoperative planning must be implemented intraoperatively with utmost precision to ensure quality and minimize intraoperative and postoperative side effects.^39^

Currently, implant treatment approaches are as follows: Free-handed implant placement, Static computer-aided implant placement, and dynamic computer-aided implant placement. The widely used free-handed implant placement provides less predictable accuracy and depends on the surgeon’s experience and expertise.^40^ Deviation in implant placement is relatively large among surgeons with different levels of experience. When novice surgeons face complex cases, achieving satisfactory results can be challenging. A systematic review^41^ based on six clinical studies indicated that the ranges of deviation of the platform, apex, and angle from the planned position with free-handed implant placement were (1.25 ± 0.62) mm–(2.77 ± 1.54) mm, (2.10 ± 1.00) mm–(2.91 ± 1.52) mm, and 6.90°± 4.40°–9.92°± 6.01°, respectively. Static guides could only provide accurate guidance for the initial implantation position. However, it is difficult to precisely control the depth and angle of osteotomies.^42^ The lack of real-time feedback on drill positioning during surgery can limit the clinician’s ability to obtain necessary information.^42–44^ Besides, surgical guides may also inhibit the cooling of the drills used for implant bed preparation, which may result in necrosis of the overheated bone. Moreover, the use of static guides is limited in patients with limited accessibility, especially for those with implants placed in the posterior area. Additionally, the use of guides cannot flexibly adjust the implant plan intraoperatively. With dynamic computer-aided implant placement, the positions of the patient and drills could be tracked in real-time and displayed on a computer screen along with the surgical plan, thus allowing the surgeon to adjust the drilling path if necessary. However, the surgeons may deviate from the plan or prepare beyond it without physical constraints. During surgery, the surgeon may focus more on the screen for visual information rather than the surgical site, which can lead to reduced tactile feedback.^45^ The results of a meta-analysis showed that the platform deviation, apex deviation, and angular deviation were 0.91 mm (95% CI 0.79–1.03 mm), 1.26 mm (95% CI 1.14–1.38 mm), and 3.25° (95% CI 2.84°–3.66°) respectively with the static computer-aided implant placement, and 1.28 mm (95% CI 0.87–1.69 mm), 1.68 mm (95% CI 1.45–1.90 mm), and 3.79° (95% CI 1.87–5.70°), respectively, with dynamic computer-aided implant placement. The analysis results showed that both methods improved the accuracy compared to free-handed implant placement, but they still did not achieve ideal accuracy.^46^ Gwangho et al.^47^ believe that the key point of a surgical operation is still manually completed by surgeons, regardless of static guide or dynamic navigation, and the human factors (such as hand tremble, fatigue, and unskilled operation techniques) also affect the accuracy of implant placement.

Robotic-assisted implant surgery could provide accurate implant placement and help the surgeon control handpieces to avoid dangerous tool excursions during surgery.^48^ Furthermore, compared to manual calibration, registration, and surgery execution, automatic calibration, registration, and drilling using the dental implant robotic system reduces human error factors. This, in turn, helps avoid deviations caused by surgeons’ factors, thereby enhancing surgical accuracy, safety, success rates, and efficiency while also reducing patient trauma.^7^ With the continuous improvement of technology and reduction of costs, implant robotics are gradually becoming available for commercial use. Yomi (Neocis Inc., USA) has been approved by the Food and Drug Administration, while Yakebot (Yakebot Technology Co., Ltd., Beijing, China), Remebot (Baihui Weikang Technology Co., Ltd, Beijing, China), Cobot (Langyue dental surgery robot, Shecheng Co. Ltd., Shanghai, China), Theta (Hangzhou Jianjia robot Co., Ltd., Hangzhou, China), and Dcarer (Dcarer Medical Technology Co., Ltd, Suzhou, China) have been approved by the NMPA. Dencore (Lancet Robotics Co., Ltd., Hangzhou, China) is in the clinical trial stage in China.

### Basic research on dental implant robotic system

Compared to other surgeries performed with general anesthesia, dental implant surgery can be completed under local anesthesia, with patients awake but unable to remain completely still throughout the entire procedure. Therefore, research related to dental implant robotic system, as one of the cutting-edge technologies, mainly focuses on acquiring intraoperative feedback information (including tactile and visual information), different surgical methods (automatic drilling and manual drilling), patient position following, and the simulation of surgeons’ tactile sensation.

#### Architecture of dental implant robotic system

**Fig. 4:**
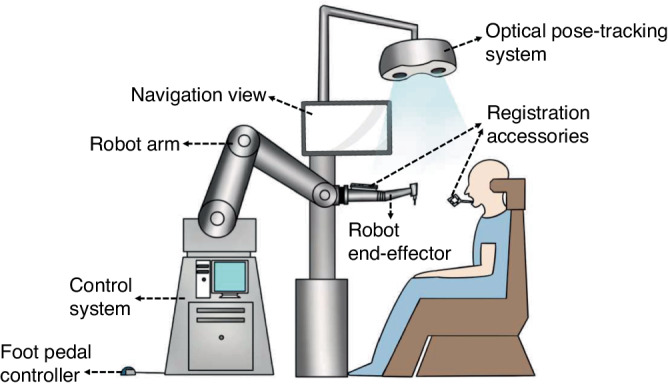
The architecture of dental implant robotics

The architecture of dental implant robotics primarily comprises the hardware utilized for surgical data acquisition and surgical execution (Fig. 4). Data acquisition involves perceiving, identifying, and understanding the surroundings and the information required for task execution through the encoders, tactile sensors, force sensors, and vision systems. Real-time information obtained also includes the robot’s surrounding environment, object positions, shapes, sizes, surface features, and other relevant information. The perception system assists the robot in comprehending its working environment and facilitates corresponding decision-making as well as actions.

During the initial stage of research on implant robotics, owing to the lack of sensory systems, fiducial markers and corresponding algorithms were used to calculate the transformation relationship between the robot’s and the model’s coordinate system. The robot was able to determine the actual position through coordinate conversions. Dutreuil et al.^49^ proposed a new method for creating static guides on casts using robots based on the determined implant position. Subsequently, Boesecke et al.^50^ developed a surgical planning method using linear interpolation between start and end points, as well as intermediate points. The surgeon performed the osteotomies by holding the handpieces, with the robot guidance based on preoperatively determined implant position. Sun et al.^51^ and McKenzie et al.^52^ registered cone-beam computed tomography (CBCT) images, the robot’s coordinate system, and the patient’s position using a coordinate measuring machine, which facilitated the transformation of preoperative implant planning into intraoperative actions.

Neocis has developed a dental implant robot system called Yomi (Neocis Inc.)^53^ based on haptic perception and connects a mechanical joint measurement arm to the patient’s teeth to track their position. The joint encoder provides information on the drill position, while the haptic feedback of handpieces maneuvered by the surgeon constrains the direction and depth of implant placement.

Optical positioning is a commonly used localization method that offers high precision, a wide -field -of -view, and resistance to interference.^54^ This makes it capable of providing accurate surgical guidance for robotics. Yu et al.^55^ combined image-guided technology with robotic systems. They used a binocular camera to capture two images of the same target, extract pixel positions, and employ triangulation to obtain three-dimensional coordinates. This enabled perception of the relative positional relationship between the end-effector and the surrounding environment. Yeotikar et al.^56^ suggested mounting a camera on the end-effector of the robotic arm, positioned as close to the drill as possible. By aligning the camera’s center with the drill’s line of sight at a specific height on the lower jaw surface, the camera’s center accurately aligns with the drill’s position in a two-dimensional space at a fixed height from the lower jaw. This alignment guides the robotic arm in drilling through specific anatomical landmarks in the oral cavity. Yan et al.^57^ proposed that the use of “eye-in-hand” optical navigation systems during surgery may introduce errors when changing the handpiece at the end of the robotic arm. Additionally, owing to the narrow oral environment, customized markers may fall outside the camera’s field of view when the robotic arm moves to certain positions.^42^ To tackle this problem, a dental implant robot system based on optical marker spatial registration and probe positioning strategies is designed. Zhao et al constructed a modular implant robotic system based on binocular visual navigation devices operating on the principles of visible light with “eye-to-hand” mode, allowing complete observation of markers and handpieces within the camera’s field of view, thereby ensuring greater flexibility and stability.^38,58^

The dental implant robotics execution system comprises hardware such as motors, force sensors, actuators, controllers, and software components to perform tasks and actions during implant surgery. The system receives commands, controls the robot’s movements and behaviors, and executes the necessary tasks and actions. Presently, research on dental implant robotic systems primarily focuses on the mechanical arm structure and drilling methods.

The majority of dental implant robotic systems directly adopt serial-linked industrial robotic arms based on the successful application of industrial robots with the same robotic arm connection.^59–62^ These studies not only establish implant robot platforms to validate implant accuracy and assess the influence of implant angles, depths, and diameters on initial stability but also simulate chewing processes and prepare natural root-shaped osteotomies based on volume decomposition. Presently, most dental implant robots in research employ a single robotic arm for surgery. Lai et al.^62^ indicated that the stability of the handpieces during surgery and real-time feedback of patient movement are crucial factors affecting the accuracy of robot-assisted implant surgery. The former requires physical feedback, while the latter necessitates visual feedback. Hence, they employed a dual-arm robotic system where the main robotic arm was equipped with multi-axis force and torque sensors for performing osteotomies and implant placement. The auxiliary arm consisted of an infrared monocular probe used for visual system positioning to address visual occlusion issues arising from changes in arm angles during surgery.

The robots mentioned above use handpieces to execute osteotomies and implant placement. However, owing to limitations in patient mouth opening, performing osteotomies and placing implants in the posterior region can be challenging. To overcome the spatial constraints during osteotomies in implant surgery, Yuan et al.^63^ proposed a robot system based on earlier research which is laser-assisted tooth preparation. This system involves a non-contact ultra-short pulse laser for preparing osteotomies. The preliminary findings confirmed the feasibility of robotically controlling ultra-short pulse lasers for osteotomies, introducing a novel method for a non-contact dental implant robotic system.

#### Position following of dental implant robotic system

It can be challenging for patients under local anesthesia to remain completely still during robot-assisted dental implant surgery.^52,64–67^ Any significant micromovement in the patient’s position can severely affect clinical surgical outcomes, such as surgical efficiency, implant placement accuracy compared to the planned position, and patient safety. Intraoperative movement may necessitate re-registration for certain dental implant robotic systems. In order to guarantee safety and accuracy during surgery, the robot must detect any movement in the patient’s position and promptly adjust the position of the robotic arm in real time. Yakebot uses binocular vision to monitor visual markers placed outside the patient’s mouth and at the end of the robotic arm. This captures motion information and calculates relative position errors. The robot control system utilizes preoperatively planned positions, visual and force feedback, and robot kinematic models to calculate optimal control commands for guiding the robotic arm’s micromovements and tracking the patient’s micromovements during drilling. As the osteotomies are performed to the planned depth, the robotic arm compensates for the patient’s displacement through the position following the function. The Yakebot’s visual system continuously monitors the patient’s head movement in real time and issues control commands every 0.008 s. The robotic arm is capable of following the patient’s movements with a motion servo in just 0.2 s, ensuring precise and timely positioning.

#### The simulation of surgeons’ tactile sensation in dental implant robotic systems

Robot-assisted dental implant surgery requires the expertise and tactile sense of a surgeon to ensure accurate implantation. Experienced surgeons can perceive bone density through the resistance they feel in their hands and adjust the force magnitude or direction accordingly. This ensures proper drilling along the planned path. However, robotic systems lack perception and control, which may result in a preference for the bone side with lower density. This can lead to inaccurate positioning compared to the planned implant position.^61,62^ Addressing this challenge, Li et al.^68^ established force-deformation compensation curves in the X, Y, and Z directions for the robot’s end-effector based on the visual and force servo systems of the autonomous dental robotic system, Yakebot. Subsequently, a corresponding force-deformation compensation strategy was formulated for this robot, thus proving the effectiveness and accuracy of force and visual servo control through in vitro experiments. The implementation of this mixed control mode, which integrates visual and force servo systems, has improved the robot’s accuracy in implantation and ability to handle complex bone structures. Based on force and visual servo control systems, Chen et al.^69^ have also explored the relationship between force sensing and the primary stability of implants placed using the Yakebot autonomous dental robotic system through an in vitro study. A significant correlation was found between Yakebot’s force sensing and the insertion torque of the implants. This correlation conforms to an interpretable mathematical model, which facilitates the predictable initial stability of the implants after placement.

During osteotomies with heat production (which is considered one of the leading causes of bone tissue injury), experienced surgeons could sense possible thermal exposure via their hand feeling. However, with free-handed implant placement surgery, it is challenging to perceive temperature changes during the surgical process and establish an effective temperature prediction model that relies solely on a surgeon’s tactile sense. Zhao et al.^70^, using the Yakebot robotic system, investigated the correlation between drilling-related mechanical data and heat production and established a clinically relevant surrogate for intraosseous temperature measurement using force/torque sensor-captured signals. They also established a real-time temperature prediction model based on real-time force sensor monitoring values. This model aims to effectively prevent the adverse effects of high temperatures on osseointegration, laying the foundation for the dental implant robotic system to autonomously control heat production and prevent bone damage during autonomous robotic implant surgery.

The innovative technologies mentioned above allow dental implant robotic systems to simulate the tactile sensation of a surgeon and even surpass the limitations of human experience. This advancement promises to address issues that free-handed implant placement techniques struggle to resolve. Moreover, this development indicates substantial progress and great potential for implantation.

### Clinical research on dental implant robotic systems

#### Clinical workflow of dental implant robotic systems

**Fig. 5:**
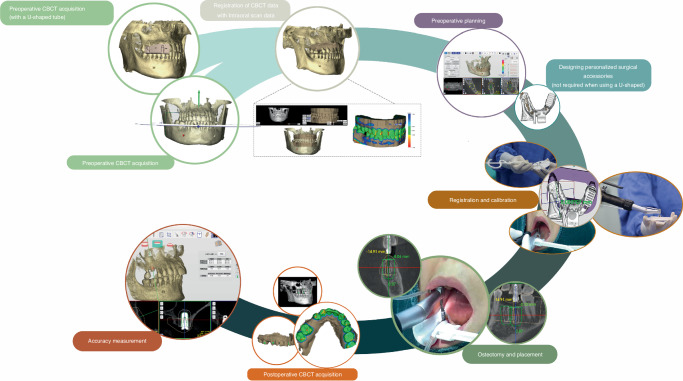
Clinical workflow of robotic-assisted dental implant placement

The robotic assistant dental implant surgery consists of three steps: preoperative planning, intraoperative phase, and postoperative phase (Fig. 5). For preoperative planning, it is necessary to obtain digital intraoral casts and CBCT data from the patient, which are then imported into preoperative planning software for 3D reconstruction and planning implant placement. For single or multiple tooth gaps using implant robotic systems (except Yakebot),^61,62,71,72^ a universal registration device (such as the U-shaped tube) must be worn on the patients’ missing tooth site using a silicone impression material preoperatively to acquire CBCT data for registration. The software performs virtual placement of implant positions based on prosthetic and biological principles of implant surgery, taking into account the bone quality of the edentulous implant site to determine the drilling sequence, insertion depth of each drill, speed, and feed rate. For single or multiple tooth implants performed using Yakebot, there is no need for preoperative CBCT imaging with markers. However, it is necessary to design surgical accessories with registration holes, brackets for attaching visual markers, and devices for assisting mouth opening and suction within the software (Yakebot Technology Co., Ltd., Beijing, China). These accessories are manufactured using 3D printing technology.

For the intraoperative phase, the first step is preoperative registration and calibration. For Yakebot, the end-effector marker is mounted to the robotic arm, and the spatial positions are recorded under the optical tracker. The calibration plate with the positioning points is then assembled into the implant handpiece for drill tip calibration. Then, the registration probe is inserted in the registration holes of the jaw positioning plate in turn for spatial registration of the jaw marker and the jaw. Robot-assisted dental implant surgery usually does not require flapped surgery,^73,74^, yet bone grafting due to insufficient bone volume in a single edentulous space or cases of complete edentulism requiring alveolar ridge preparation may require elevation of flaps. For full-arch robot-assisted implant surgery, a personalized template with a positioning marker is required and should be fixed with metallic pins for undergoing an intraoperative CBCT examination, thus facilitating the robot and the jaws registration in the visual space and allowing the surgical robot to track the patient’s motion. The safe deployment of a robot from the surgical site is an essential principle for robot-assisted implant surgery. In the case of most robots, such as Yomi, the surgeon needs to hold the handpieces to control and supervise the robot’s movement in real time and stop the robotic arm’s movement in case of any accidents. With Yakebot, the entire surgery is performed under the surgeon’s supervision, and immediate instructions are sent in response to possible emergencies via a foot pedal. Additionally, the recording of the entrance and exit of the patient’s mouth ensures that the instruments would not damage the patient’s surrounding tissues. The postoperative phase aims at postoperative CBCT acquisition and accuracy measurement.

In clinical surgical practice, robots with varying levels of autonomy perform implant surgeries differently. According to the autonomy levels classified by Yang et al.^6,8,33^ for medical robots, commercial dental implant robotic systems (Table 2) currently operate at the level of robot assistance or task autonomy.

**Table 2:** The autonomous level of commercial dental implant robotics

Robotic platform	Country	Autonomy level	Perception system	Robot arm	Reference
Yomi	USA	Level1: robot assistance	Haptic guidance	Unpublished	^53,55^
Theta	China	Level1: robot assistance	Two-eye infrared camera	UR3e	^62,76^
Dcarer	China	Level1: robot assistance	Two-eye infrared camera	UR5	^81^
Cobot	China	Level1: robot assistance	Haptic guidance and single-eye infrared camera	UR3 (main arm) and micro infrared single-eye tracking probe (auxiliary arm)	^62^
Remebot	China	Level2: task autonomy	Two-eye visible camera	UR5	^76,82^
Yakebot	China	Level2: task autonomy	Two-eye infrared camera	UR5	^70,73,79,80^

The robot-assistance dental implant robotic systems provide haptic,^75^ visual or combined visual and tactile guidance during dental implant surgery.^46,76,77^ Throughout the procedure, surgeons must maneuver handpieces attached to the robotic guidance arm and apply light force to prepare osteotomies.^62^ The robotic arm constrains the 3D space of the drill as defined by the virtual plan, enabling surgeons to move the end of the mechanical arm horizontally or adjust its movement speed. However, during immediate implant placement or full-arch implant surgery, both surgeons and robots may struggle to accurately perceive poor bone quality, which should prompt adjustments at the time of implant placement. This can lead to incorrect final implant positions compared to the planned locations.

The task-autonomous dental implant robotic systems can autonomously perform partial surgical procedures, such as adjusting the position of the handpiece to the planned position and preparing the implant bed at a predetermined speed according to the pre-operative implant plan, and surgeons should send instructions, monitor the robot’s operation, and perform partial interventions as needed. For example, the Remebot^77,78^ requires surgeons to drag the robotic arm into and out of the mouth during surgery, and the robot automatically performs osteotomies or places implants according to planned positions under the surgeon’s surveillance. The autonomous dental implant robot system, Yakebot,^73,79,80^ can accurately reach the implant site and complete operations such as implant bed preparation and placement during surgery. It can be controlled by the surgeon using foot pedals and automatically stops drilling after reaching the termination position before returning to the initial position. Throughout the entire process, surgeons only need to send commands to the robot using foot pedals.

#### Clinical performance of robot-assisted implant surgery

**Fig. 6:**
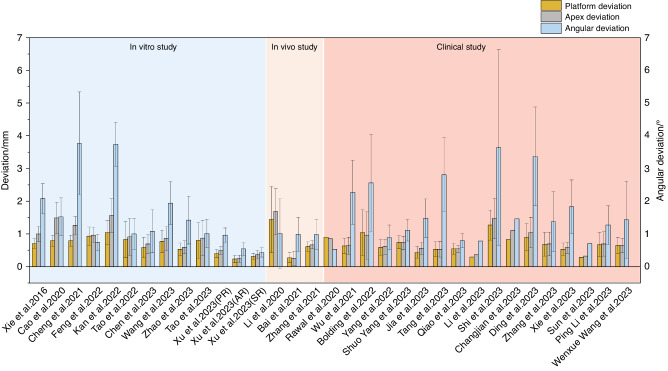
Accuracy reported in studies on robotic-assisted implant placement

Figure 6 shows the results of accuracy in vitro, in vivo, and clinical studies on robot-assisted implant surgery.^20,46,48,55,62,64,67–72,75–89^ The results suggest that platform and apex deviation values are consistent across different studies. However, there are significant variations in angular deviations among different studies, which may be attributed to differences in the perception and responsiveness to bone quality variances among different robotic systems. Therefore, future development should focus on enhancing the autonomy of implant robots and improving their ability to recognize and respond to complex bone structures.

**Fig. 7:**
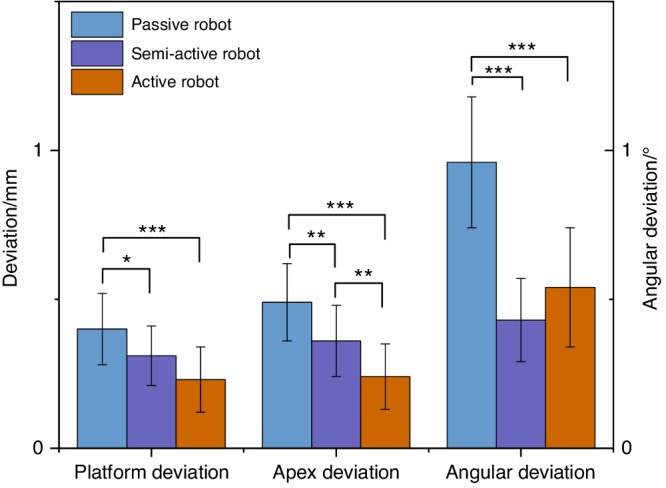
Comparison of accuracy of dental implant robotics with different levels of autonomy (phantom experiments) (*P < 0.05, **P < 0.01, ***P < 0.001)

Xu et al.^77^ conducted a phantom experimental study comparing the implant placement accuracy in three levels of dental implant robotics, namely passive robot (Dcarer, level 1), semi-active robot (Remebot, level 2), and active robot (Yakebot, level 2) (Fig. 7). The study found that active robot had the lowest deviations at the platform and apex of the planned and actual implant positions, While the semi-active robot also had the lowest angular deviations. Chen et al.^46^ and Jia et al.^79^ conducted clinical trials of robotic implant surgery in partially edentulous patients using a semi-active dental implant robotic system (level 1) and an autonomous dental implant robot (level 2). The deviations of the implant platform, apex, and angle were (0.53 ± 0.23) mm/(0.43 ± 0.18) mm, (0.53 ± 0.24) mm/(0.56 ± 0.18) mm and 2.81° ± 1.13°/1.48° ± 0.59°, respectively. These results consistently confirmed that robotic systems can achieve higher implant accuracy than static guidance and that there is no significant correlation between accuracy and implant site (such as anterior or posterior site). The platform and angle deviation of autonomous dental implant robots were smaller than those of semi-active dental implant robotic systems. Li et al.^73^ reported the use of the autonomous dental implant robot (level 2) to complete the placement of two adjacent implants with immediate postoperative restoration. The interim prosthesis fabricated prior to implant placement was seated without any adjustment, and no adverse reactions occurred during the operation.

**Fig. 8:**
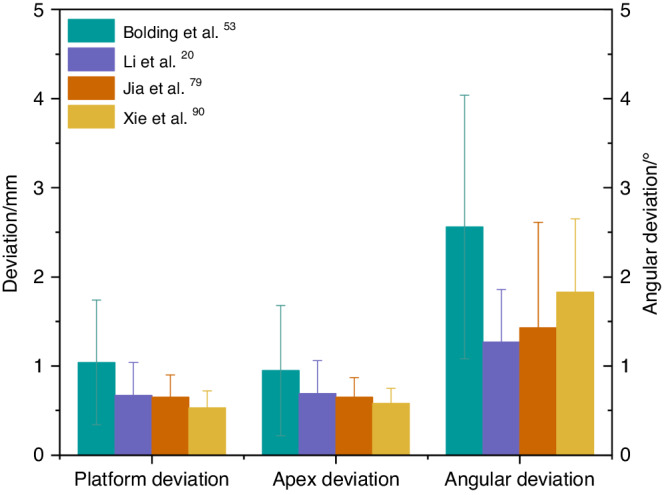
Comparison of accuracy in robotic-assisted full-arch implant placement

Bolding et al.,^53^ Li et al.,^20^ Jia et al.,^79^ and Xie et al.^90^ used dental implant robots to conduct clinical trials in full-arch implant surgery with five or six implants placed in each jaw. The deviations of implant platform, apex, and angle are shown in Fig. 8. The haptic dental implant robot (level 1) used by Bolding et al.,^53^ achieved more deviations compared to other studies that used semi-active (level 1) or active robots (level 2). As its handpiece must be maneuvered by the surgeon, human errors such as surgeon fatigue may not be avoided. Owing to the parallel common implant placement paths between various implant abutments, prefabricated temporary dentures could be seated smoothly, and some patients wore temporary complete dentures immediately after surgery. These results indicate that robotic systems can accurately locate and perform implant placement during surgery.

**Fig. 9:**
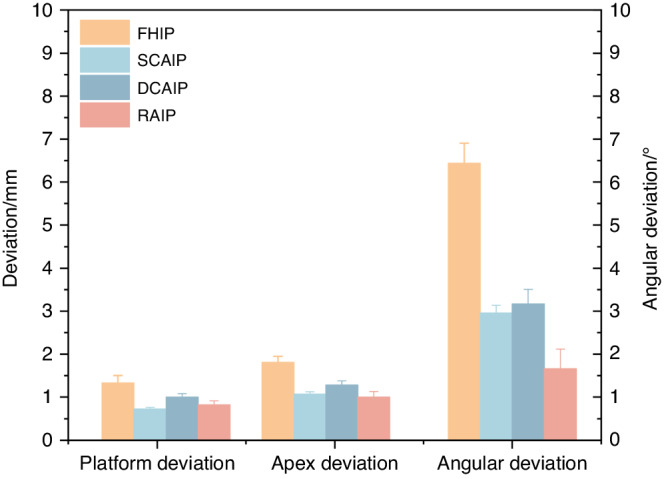
Comparison of accuracy of free-handed, static, dynamic, and robotic-assisted implant placement. (FHIP free-hand implant placement, SCAIP static computer-aided implant placement, DCAIP dynamic computer-aided implant placement, RAIP robot-assisted implant placement)

As there are relatively few studies of implant robots in clinical applications, Tak ´acs et al.^91^ conducted a meta-analysis under in vitro conditions with free-handed, static-guided, dynamic navigated, and robotic-assisted implant placements, as shown in Fig. 9. It was found that, compared to free-handed, static guided and dynamic navigated implant placements, robotic-assisted implant placements have more advantages in terms of accuracy. However, in vitro studies cannot fully simulate the patients’ oral condition and bone quality. Recent clinical studies^89,92,93^ have shown a lower deviation in robotic-assisted implant placements compared to static-guided and dynamic-navigated implant placements. Common reasons for deviations in static-guided and dynamic-navigated implant placements include the following: deflection caused by hand tremors due to dense bone during surgery, surgeons’ experience, and other human factors. Larger clinical studies will be needed in the future to evaluate the differences between robotic and conventional surgical approaches and to provide guidance for the further development and refinement of robotic techniques.

For the long-term follow-up performance of robotic systems used in dental implant procedures, none of the comparative studies was longer than a year. One 1-year prospective clinical study by Xie et al.^90^ showed that the peri-implant tissues after robot-assisted full arch surgery at 1-year visit remained stable. There is little evidence indicating clinical outcomes especially for patient-reported outcomes. A more detailed clinical assessment should be included for further research.

### Current issues with dental implant robotic systems

#### Need for further simplification of robotic surgical procedures

Although robotic-assisted dental implant surgery can improve accuracy and treatment quality,^94^ it involves complex registration, calibration, and verification procedures that prolong the duration of surgery. These tedious processes may introduce new errors,^61^ and lower work efficiency, especially in single tooth implant placement^62^ that could extend visit times and affect patient satisfaction.^62^ Besides, surgeons are required to undergo additional training to familiarize themselves with the robotic system.^87^

#### Need for improved flexibility of dental implant robotic system

During implantation, the drill tips at the end of the robotic arms cannot be tilted, and this can increase the difficulty of using robots in posterior sections with limited occlusal space.^61,62^ In addition, currently available marker systems require patients to wear additional devices to hold the marker in place. If these markers are contaminated or obstructed by blood, the visual system may not be able to detect them, limiting surgical maneuverability to some extent. During immediate implant placement or in cases of poor bone quality in the implant site, the drill tips may deviate towards the tooth sockets or areas of lower bone density, seriously affecting surgical precision.

Currently, only one study has developed a corresponding force-deformation compensation strategy for robots,^68^ but clinical validation is still lacking. Additionally, the dental implant robotic system, along with other dental implant robots developed for prosthetics, endodontics, and orthodontics, is currently single-functional. Multi-functional robots are required for performing various dental treatments.

#### Difficulties in promoting the use of dental implant robotic system

Despite the enormous potential of robotic systems in the medical field, similar to the development of computer-aided design/computer-aided manufacturing technology, introducing and applying this technology faces multiple challenges in the initial stages. The high cost of robotic equipment may limit its promotion and application in certain regions or medical institutions. Surgeons require specialized technical training before operating robotic systems, which translates to additional training costs and time investment.^95^

## Prospects in the use of dental implant robotic system

Medical robots possess high-precision sensing and positioning capabilities, which enable precise operations at small scales. They are also equipped with safety mechanisms and stability controls to ensure the safety of medical procedures and reduce risks to patients. As technology evolves, hardware and algorithms are continuously updated, resulting in constant performance improvements. Today, medical robots are widely used in surgery, diagnosis, and rehabilitation.^7^ They enable precise and minimally invasive operation, thus reducing patient trauma and pain, shortening hospitalization, and speeding recovery, as well as reducing the need for re-operations and blood transfusions.^96^ In addition, medical robots can reduce radiation exposure for both surgeons and patients. By leveraging machine learning and artificial intelligence technologies, robots can provide personalized and intelligent treatment plans and recommendations based on large amounts of data, improving diagnostic efficiency. Robots with remote operation capabilities can enable remote surgeries or consultations across regions, facilitating access to medical services. Moreover, robots can work continuously, ensuring medical quality and consistency while reducing surgeons ’neck and back pain,^97^ as well as numbness in the hands and wrists experienced by surgeons.^98^ Besides, they also reduce mental and physical stress, improving surgeons’ quality of life and extending their career longevity.

From da Vinci surgical robotic system to dental implant robotic system, these innovative technologies are leading unprecedented changes in the medical field. Dental implant robotic system continuously improves software modules and optimizes operating procedures to become more intelligent, more flexible and easier to learn and use. In the future, more extensive clinical trials will be needed to continuously observe and evaluate the long-term outcomes of robot-assisted implant surgery, especially in multi-center clinical trials. Moreover, measured outcomes must include well-defined clinical outcomes (such as pathophysiology^99^), technical outcomes (including those derived from robotic kinematic and haptic sensors^100^), patient-reported outcomes (such as quality-of-life indicators and overall satisfaction with treatment^99^), and wider outcomes that reflect potential robotic disruption (ergonomic benefits, impacts on accessibility to surgery^100^) where relevant. In addition, the evaluation of dental implant robots requires the analysis of learning curves. Large prospective cohorts provide the first opportunity to capture real-world learning curves, which can be used to develop training mechanisms that shorten learning curves and minimize any negative impact on patients.^99,100^

As a pioneering attempt, the dental implant robotic system provides an important exploration and paradigm for the application of another dental robotic system. As technology continues to advance, robotics and artificial intelligence will provide more precise diagnostic and treatment options, more intelligent medical decision support systems, as well as more flexible and precise surgical procedures. These revolutionary technologies will continue to drive advances in medicine and healthcare, opening up new possibilities for future clinical practice.

## Conclusion

With novel technology advancements, medical robotics are bringing a new era to medicine. Innovative medical robotics can perform surgical procedures, aid rehabilitation, make diagnoses, achieve robotic laboratory automation and other robots suitable for various functional needs. In the field of dentistry, the most widely utilized robotic system presently is the dental implant robotic system. Implant robotic systems could offer a more flexible approach for the precise planning, and visual and haptic guidance of surgical procedures. Various clinical trials have confirmed the high accuracy of implant robotic-assisted surgery achieved and toward long-term implant success. However, there is still much room for improvement in terms of further simplification, the flexibility of robotic surgical procedures, and systematic education. By leveraging machine learning and artificial intelligence technologies, more precise diagnostic and treatment options, intelligent medical decision support systems, and flexible and precise surgical procedures will be provided for future clinical practice.
